# Meaning of home attenuates the relationship between functional limitations and active aging

**DOI:** 10.1007/s40520-024-02810-x

**Published:** 2024-08-01

**Authors:** Björn Slaug, Magnus Zingmark, Marianne Granbom, Jonas Björk, Taina Rantanen, Steven M. Schmidt, Susanne Iwarsson

**Affiliations:** 1 Department of Health Sciences, Faculty of Medicine, Lund University, Lund, Sweden; 2 Health and Social Care Administration, Municipality of Östersund, Östersund, Sweden; 3 Community Medicine and Rehabilitation, Umeå University, Umeå, Sweden; 4 Clinical Studies Sweden, Forum South, Skåne University Hospital, Lund, Sweden; 5 Division of Occupational and Environmental Medicine, Lund University, Lund, Sweden; 6 Faculty of Sport and Health Sciences, Gerontology Research Centre, University of Jyväskylä, Jyväskylä, Finland

**Keywords:** Home, Housing choices, Moving, Prospective study, Residential choices

## Abstract

**Background and aims:**

Active aging is the process through which people strive to maintain wellbeing when growing old. Addressing the lack of research on active aging in the context of housing, the aim was to describe active aging among people aged 55 and older considering relocation and investigate whether perceived housing moderates the relationship between functional limitations and active aging.

**Methods:**

We utilized cross-sectional data from a sub-sample (*N* = 820; mean age = 69.7; 54% women) of the Prospective RELOC-AGE. Functional limitations were reported using 10 dichotomous questions. Active aging was assessed with the University of Jyvaskyla Active Aging Scale (UJACAS; 17 items, self-rated for four perspectives). Perceived housing was self-rated with four usability questions and meaning of home (MOH; 28 items). Cross-sectional associations and interactions were analysed using linear regression models, adjusting for gender and educational level.

**Results:**

Each functional limitation decreased the active aging score by almost five points (*p* < 0.001). Usability did not moderate that relationship while MOH significantly attenuated the association between functional limitations and active aging (*p* = 0.039). Those with high MOH had two points less decrease in active aging score compared to those with low MOH.

**Discussion and conclusions:**

Having a home with more personal meaning attached to it seems to provide more ability and opportunity for meaningful activities, thus supporting active aging despite functional limitations. This sheds new light on the known association between MOH and different aspects of wellbeing in old age and has relevance for theory development, housing policies and housing counselling targeting younger older adults.

## Introduction

The home is the predominant context for activities as we age, and housing circumstances may support or hinder participation in various activities promoting health and wellbeing. Outlined already in the much-cited Disablement Process Model [1], it is well established that functional limitations are associated with activity performance and mobility-related disability [2, 3]. When functional limitations increase with age, perceived qualities such as usability or meaning of the home as well as objective housing qualities may influence the degree of activity among older people. Such dynamics are well known [see e.g., 4] and have been described based on empirical research involving very old adults [5]. However, because conceptualizations as well as empirical studies on aging and housing predominantly focus on phases of the aging process where needs for care and special housing emerge [e.g., 6, 7], the knowledge about dynamics of housing and aging among younger and healthier older adults (aged < 75 years) is insufficient.

### Active aging

The concept of active aging stems from the work of Robert Havighurst [8] who suggested that staying active in later life results in wellbeing. Framed as a policy goal, more than 20 years ago, the World Health Organization defined active aging as the process of optimizing opportunities for health, participation, and security to enhance quality of life as people age and stated that policies and programs should be based on the rights, needs, preferences and capacities of older people [9]. This goal is currently reflected in the actions and programs of many countries and organizations. However, the empirical research on active aging in terms of how older individuals live their everyday lives has remained obscure with sometimes normative and often varied approaches. Addressing the individual level of active aging, Rantanen et al. [10] put forward the following definition: Active aging refers to the striving for activities relating to a person’s goals, functional capacities and opportunities. Based on this, a validated self-report scale encompassing goals, activity, ability and opportunity in different life areas with a wide range of activities was launched (ibid.). While intact mobility often coincides with higher active aging scores [11], people with reduced community mobility may also maintain high levels of active aging by concentrating on activities that may be done at home [12]. From a gender perspective, it is also important to consider activities that are not primarily based on the work and retirement trajectory that tends to favor men [13]. Because higher active aging scores strongly correlates with better quality of life [14], gaining a better understanding of how environmental factors related to housing arrangements can promote active aging in a more equitable manner, could be useful for the development of age-friendly policies.

A recent study compared older adults aged 75 + in different forms of housing in Finland, using the active aging scale mentioned [15]. Reflecting that those living in special forms of housing usually have more functional limitations, activity limitations and participation restrictions, Siltanen et al. [15] reported that men living in senior housing had lower levels of active aging than men in ordinary housing. As to women, those living in senior housing demonstrated a greater will to be active but had poorer ability and possibilities for activity than those in ordinary housing.

### Perceived aspects of housing

Perceived aspects of housing refer to the totality of subjective phenomena of experiences and symbolic representations related to living at home [16]. Objective aspects of housing (e.g., accessibility) are important as well, but foremost among people with lower levels of functioning [17]. There is some research on perceived aspects of housing (e.g., usability, meaning of home) using established instruments for self-assessments among older adults aged 75+ (e.g., 18], younger older people [19, 20], and in diagnosis-specific cohorts [21]. As to younger and healthier older adults, Kylén et al.’s study [20] shows that perceived aspects of housing are associated with psychological well-being, which is an indication that it is fruitful to address perceived housing to increase the knowledge about active aging in the context of housing in younger cohorts.

Usability of the home was conceptualized integrating occupational therapy theory [22] such as the person-environment-occupation model [23] with the Ecological Theory of Aging [24]. That is, activity performance at home is shaped by the transaction between person, environment and occupation. Usability is a perceived aspect of housing addressing the extent to which housing needs and preferences can be fulfilled in terms of activity performance at home, as perceived by the individual [25]. That is, the focus is on daily activity and the perceived functionality of the dwelling, addressing self-assessed possibilities to perform necessary and preferred activities in a specific home environment.

Theoretically underpinned by psychology and environmental gerontology [26, 27], the concept meaning of home (MOH) denotes a form of place attachment by symbolic representation of space, place and personal meaning tied to the home [28], suggesting that the home is not just an objective function but related to individual experiences. The complex concept is rooted in “what makes the house a home” and focuses on the relationship among the socio-physical setting of the home and subjective evaluations, values, emotions, and goals. Results from a study among very old people living in ordinary housing indicate that those who report higher meaning of home are more independent in daily life and have higher psychological well-being [18], and similar findings regarding psychological well-being were found among people aged 67–70 years [20].

### Rationale and study aim

As people approach and pass retirement age it is common to start pondering about one’s housing situation. Retirement, perceptions of oneself as aging and the transition from working life to a markedly changed everyday life elicit thoughts about how and where to live [29]. Although the importance of the home for active aging has been highlighted for instance by Bronswijk [30], to the best of our knowledge no empirical findings regarding perceived aspects of housing and active aging on the individual level among younger older adults living in ordinary housing and anticipating changes to their future housing situation have been reported in the scientific literature. Developing such knowledge derived from studies targeting adults at early stages of the aging process has the potential to nurture the evidence base for proactive housing policies, health promotion and interventions. With that perspective in mind, this study explored whether positive perceptions of the home can moderate the expected negative impact of functional limitations on active aging. The aim was to describe self-reported active aging in a sample of people aged 55 years or older and investigate whether usability in the home and meaning of home, respectively, moderate the relationship between functional limitations and active aging.

## Methods

We used baseline data from the Prospective RELOC-AGE ongoing longitudinal two-level mixed-method cohort study in Sweden, collected in 2021. For details, see study protocol [28]; International Registered Report Identifier [IRRID]: DERR1-10.2196/31137. Prospective RELOC-AGE was registered at ClinicalTrials.gov NCT04765696. For the current study, we used a cross-sectional design, utilizing the baseline data.

### Population and recruitment

The procedures were designed to recruit an information-rich sample to explore housing choices, relocation, and active aging from an early stage in the aging process. We included people aged 55 years or older, with a postal address in Sweden and listed with an interest in relocation at either of three housing companies: two municipal public housing companies and one national provider of tenant-owned dwellings. In this way, residents from a diversity of types of apartments in multi-family housing typically attracting people from different socio-economic groups were represented. Severe cognitive impairment or insufficient language skills to give informed consent or participate in telephone interviews were exclusion criteria.

The recruitment period lasted from March to December 2021. During the first stage of recruitment, contact information for persons on two of the housing companies’ interest lists were delivered to the university following the companies’ procedures to ensure handling according to European General Data Protection Regulations (GDPR). During the first phase of recruitment, 6,977 people were invited to participate. However, due to a GDPR incident within one of the companies, we had to implement a process to safeguard legal requirements. Following this, the recruitment process was revised so that persons on the company’s interest lists were informed by a newsletter from the company about the opportunity to sign up via an Internet page set up by the research team on the university’s website. In this recruitment phase, the third housing company was included to expand the recruitment base. Taken together, an additional 1,606 persons listed their interest to participate. In all, the 1,964 persons who agreed to participate received an invitation letter by postal mail including information about the study, procedures, handling of data, and information on how to access a web survey via an online portal. One reminder was sent to those who had listed their interest to participate. The present study was based on data collected with a subsample (*n* = 820) who volunteered to participate in additional data collection by telephone (described under Data collection). Sample characteristics are presented in Table 1.

**Table 1:** Sample characteristics, *N* = 820

	Functional limitations = 0	Functional limitations = 1	Functional limitations > 1	Total
	*n* = 380	*n* = 180	*n* = 260	*N* = 820
Variable	Mean (SD) or column % (n)			
Age	68.3 (7.3)	69.3 (6.9)	71.9 (8.2)	69.7 (7.7)
Gender				
Men	52.4 (199)	45.6 (82)	47.7 (124)	49.4 (405)
Women	47.6 (181)	54.4 (98)	52.3 (136)	50.6 (415)
Civil status ^a^				
Married/registered partner	67.1 (255)	68.9 (122)	64.3 (166)	66.2 (543)
Country of origin ^b^				
Sweden	92.3 (347)	94.9 (169)	93.4 (240)	93.2 (756)
Other	7.7 (29)	5.1 (9)	6.6 (17)	6.8 (55)
Education ^c^				
Elementary school	5.0 (19)	3.4 (6)	12.0 (31)	6.9 (56)
2 years upper secondary school	9.0 (34)	5.6 (10)	9.7 (25)	8.5 (69)
3 or 4 years upper secondary school	13.7 (52)	16.3 (29)	12.7 (33)	14.0 (114)
University less than 3 years	19.8 (75)	16.3 (29)	21.2 (55)	19.5 (159)
University 3 years or more	52.5 (199)	58.4 (104)	44.4 (115)	51.2 (418)
Type of housing ^d^				
Single-family house	49.9 (188)	49.4 (89)	50.0 (130)	49.8 (407)
Apartment in multi-dwelling block	49.6 (187)	50.0 (90)	50.0 (130)	49.8 (407)
Other	0.5 (2)	0.6 (1)	0.0 (0)	0.4 (3)
Housing tenure ^e^				
Privately owned	87.4 (326)	81.5 (145)	86.1 (223)	85.7 (694)
Rented	12.6 (47)	18.5 (33)	13.9 (36)	14.3 (116)
Type of area ^a^				
Urban	63.7 (240)	53.9 (97)	59.7 (154)	60.2 (491)
Semi-urban	28.4 (107)	35.6 (64)	29.8 (77)	30.4 (248)
Rural	8.0 (30)	10.6 (19)	10.5 (27)	9.3 (76)
Stairs ^f^	70.2 (262)	72.1 (129)	65.8 (169)	69.2 (560)
Ramp/elevator ^g^	32.5 (120)	29.6 (53)	33.7 (87)	32.3 (260)
Number of persons in the household ^a^	1.87 (0.65)	1.83 (0.68)	1.78 (0.63)	1.83 (0.65)
SF-36, General health ^h^	3.96 (0.80)	3.47 (0.88)	2.84 (0.89)	3.50 (0.97)
Poor	0.0 (0)	0.6 (1)	2.7 (7)	1.0 (8)
Fair	2.4 (9)	11.7 (21)	37.7 (98)	15.3 (128)
Good	26.6 (101)	39.7 (71)	35.4 (92)	32.2 (264)
Very good	43.9 (167)	35.8 (64)	21.2 (55)	34.9 (286)
Excellent	27.1 (103)	12.3 (22)	3.1 (8)	16.2 (133)

^a^ missing = 5. ^b^ missing = 9. ^c^ missing = 4. ^d^ missing = 3. ^e^ missing = 10. ^f^ missing = 11. ^g^ missing = 14. ^h^ missing = 1

### Ethics

Following the principles of the Helsinki Declaration and current national legislation and policies on ethics for research involving humans, Prospective RELOC-AGE was approved by the Swedish Ethical Review Authority (No. 2020–03457). Information mailed to potential participants stressed that participation was voluntary, and participants had the right to decline without any consequences regarding housing offers and societal services. Informed consent was considered confirmed when filling out and submitting the survey responses.

### Data collection

Data were collected through a web-based survey and a telephone interview. The survey included established instruments for studies on aging and housing as well as study-specific questions. Due to their complex nature, three instruments (of which two are core instruments for the present study) were administered during a telephone interview with participants who agreed to this additional data collection (*n* = 820). The telephone interviews were conducted by occupational therapists and occupational therapy students trained for the task, and each telephone interview took on average 22 min. For details, see [31].

### Descriptive data

Demographic variables included age, civil status, country of origin, gender, and education. Descriptive health data included self-rated health evaluated with the one-item question from the SF-12 scale [32]; “In general, would you say your health is…” (five response options from poor to excellent). Descriptive housing variables included type of area (urban, semi-urban or rural), type of dwelling (single-family house or apartment in multi-dwelling block), type of tenure (rented or owner occupied), if there were stairs, ramp, or elevator at the entrance (yes/no), number of people in the household, and year moved to present dwelling.

### Active aging – dependent variable

Active aging was evaluated with the University of Jyvaskyla Active Aging Scale (UJACAS) [10] and was administered during the telephone interview. The instrument includes 17 activities: practicing memory, using a computer, advancing matters in one’s own life, exercising, enjoying the outdoors, taking care of one’s personal appearance, crafting or do-it-yourself, making one’s home cozy and pleasant, helping others, maintaining friendships, getting to know new people, balancing personal finances, making one’s days interesting, practicing artistic hobbies, participating in events, advancing societal/communal matters, and doing things in accord with one’s world view. Each activity is self-rated from four perspectives: *Goals* (to what extent the person wants to do the activity), *Ability* (to what extent the person is able to do it), *Opportunity* (to what extent the person perceives opportunities to do it), and *Activity* (how often or how much the person does it) on a Likert scale ranging from 0 to 4 (higher = more active aging), with respect to the past 4 weeks. The response options are tailored to suit the activity, for example, from “not at all” to “daily or almost daily” for Activity and from “not at all” to “very strongly” for Goals. Sub-scores are summed for the four perspectives Goals, Ability, Opportunity, Activity (range 0–68) and for a Total active aging score (range 0–272) that is calculated by adding up the four sub-scores. In the current study, the total score was used in the analyses of potential moderating influences on the relationship between functional limitations and active aging. The total score was computed if there were at most two missing values for each sub-score. The following formula was used to impute missing data: (sum score/items responded to) x items offered. The reliability and validity of the UJACAS were considered satisfactory for research purposes (ICC = 0.88; Cronbach’s α = 0.91) [10].

### Functional limitations – independent variable

Ten cognitive, motor and sensory functional limitations (i.e., in cognition, sight, hearing, balance, coordination, stamina, moving head, upper extremities, fine motor control, lower extremities) were self-rated (present/not present) using items adapted from the person component of the Housing Enabler instrument [33]. The variable used was the total number of functional limitations (0–10).

### Usability in the home and meaning of home - moderating variables

Usability in the home was evaluated with four items from the Usability in My Home (UIMH) instrument [25]. The respondent rated to what extent he/she perceived the current dwelling was adequately designed for managing 1/ basic activities of daily living (e.g., bathing, toileting); 2/ food preparation; 3/ washing, cleaning and flower care; 4/ laundry and grooming (scale ranging from 1 to 5; higher = more usable). A mean score was calculated for the four items, allowing at most one missing value.

MOH was evaluated with the Meaning of Home Questionnaire, which was administered during the telephone interview. The questionnaire includes 28 statements concerning four domains (physical, behavioural, cognitive/emotional and social). For example, the physical domain included statements such as “Being at home means for me living in a place that is comfortable and tastefully furnished”; the behavioural domain “Being at home means for me doing everyday tasks”; the cognitive/emotional domain “Being at home means for me feeling safe”; and the social domain “Being at home means for me meeting family, friends and acquaintances”. The respondent rates each item from 0 (strongly disagree) to 10 (strongly agree). For the present study we used the mean score for all items (range 0–10, higher score = more meaning) [16]. The instrument has adequate psychometric properties for research use with adults aged 67–70 in Sweden [19].

### Statistical analyses

Sample characteristics were presented using descriptive statistics. The relationship between functional limitations and active aging (UJACAS) was modelled using linear regression, with active aging as the dependent variable and number of functional limitations as the independent variable. Similarly, linear regression was conducted to analyze relationships between usability and MOH with active aging. To explore the moderating influence of usability and MOH respectively, two different interaction models for each variable were analyzed, one unadjusted and one adjusted for gender and education. Due to a skewed distribution of usability (66% had total score = 5), this variable was dichotomized (cutoff < 5) in the analysis, and the effect of functional limitations on active aging is presented for both levels (high/low) of usability. MOH was treated as a continuous variable in all analyses, but to visualize the moderating influence, we present the effect of functional limitations at three different levels of MOH, that is 1/ mean minus one standard deviation, 2/ mean, and 3/ mean plus one standard deviation.

P-values < 0.05 were considered statistically significant. All analyses were performed with SPSS Statistics 28 for Windows.

## Results

The number of functional limitations averaged just above one (mean = 1.3, SD = 1.8), and 46% had no functional limitations at all. Those with lowest education averaged two functional limitations, while those with highest education averaged one. Usability and MOH were scored high in the scales across education levels and genders. See Table 2 for details.

**Table 2:** Descriptives of active aging, and independent and moderating variables used in the regression analyses, *N* = 820

	Education							Gender			Total
	University > = 3 years *n* = 417	University < 3 years *n* = 159	3–4 years upper second. school *n* = 114		2 years upper second. school *n* = 69	Elementary school *n* = 56		Men *n* = 405	Women *n* = 415		*N* = 820
	Mean (SD)										
*Independent variable*											
No. of functional limitations	1.1 (1.6)	1.4 (1.8)	1.3 (1.7)		1.6 (2.2)	2.0 (2.0)		1.3 (1.7)	1.3 (1.8)		1.3 (1.8)
*Moderating variable*											
Usability ^a^	4.7 (0.6)	4.8 (0.4)	4.7 (0.6)		4.8 (0.4)	4.8 (0.4)		4.8 (0.5)	4.7 (0.6)		4.7 (0.7)
MOH ^b^	8.2 (0.9)	8.3 (0.7)	8.3 (0.8)		8.6 (0.6)	8.4 (0.8)		8.2 (0.8)	8.4 (0.8)		8.3 (0.8)
*Dependent variable*											
Active aging scores ^c^											
Goals	49.7 (8.4)	49.0 (8.1)	47.6 (8.5)		46.9 (7.7)	43.5 (8.4)	46.6 (8.2)		50.6 (8.3)	48.6 (8.5)	
Ability	62.5 (6.5)	61.8 (7.2)	62.2 (6.5)		62.9 (5.8)	59.7 (5.9)	61.9 (6.3)		62.4 (6.6)	62.1 (6.4)	
Opportunity	55.5 (9.6)	54.6 (9.9)	54.5 (8.8)		55.0 (9.3)	51.3 (8.5)	55.3 (8.9)		54.4 (10.1)	54.8 (9.5)	
Activity	43.3 (8.7)	42.7 (7.9)	42.2 (8.7)		41.6 (6.3)	39.4 (8.4)	41.8 (8.2)		43.5 (8.6)	42.7 (8.4)	
Total score	211.0 (24.9)	208.2 (24.3)	206.5 (26.1)		206.3 (19.9)	194.0 (23.4)	205.6 (24.4)		210.9 (25.3)	208.3 (24.9)	

Missing values: Usability = 2, MOH = 17, UJACAS = 4. ^a^ Usability scores [23] range from 1 to 5; higher score = better usability. ^b^ MOH scores [15] range from 1 to 10, higher score = more meaning. ^c^ UJACAS [10] sub-scores range from 0 to 68, and total score ranges from 0 to 272, higher score = more active aging

Initial univariable analyses with the active aging score as the dependent variable showed that the number of functional limitations had a significant (*p* < 0.001) negative association with active aging (β -4.6, 95% CI -5.5; -3.7), and usability (β 5.4, 95% CI 1.8; 9.0) and MOH (β 2.4, 95% CI 2.0; 2.9) had significant positive associations (*p* = 0.003 and *p* < 0.001, respectively) with active aging. In subsequent unadjusted multivariable analyses of moderating influences that included the number of functional limitations, usability was not significantly associated with active aging (*p* = 0.593) and there was no moderating influence of usability (*p* = 0.275) on active aging. Adjusting for gender and education (both significant) did not change the outcome, as the moderating influence of usability on active aging was still not significant (*p* = 0.342), see Table 3.

**Table 3:** Moderating influence of usability in my home ^a^ on the relationship between functional limitations and active aging ^b^, analysed using linear regression models, *N* = 820

Moderating influence of usability, dependent variable active aging			
Unadjusted model	Regression coefficient (β)	95% CI	P-value
No. of functional limitations	-5.1	-6.4; -3.7	< 0.001
Usability	1.2	-3.2; 5.6	0.593
Interaction: Usability*No. of functional limitations	1.0	-0.8; 2.9	0.275
R^2^ = 0.11			
Adjusted model			
No. of functional limitations	-4.7	-6.1; -3.3	< 0.001
Usability	1.6	-2.7; 5.9	0.470
Interaction: Usability*No. of functional limitations	0.9	-1.0; 2.8	0.342
Gender (Ref: Woman)	-4.8	-8.0; -1.5	0.004
Education (Ref: University 3 years or more)			
Education Elementary school	-12.3	-18.9; -5.7	< 0.001
Education 2 years upper secondary school	-2.0	-8.1; 4.0	0.504
Education 3 or 4 years upper secondary school	-3.4	-8.3; 1.4	0.167
Education University less than 3 years	-1.5	-5.8; 2.8	0.484
R^2^ = 0.13			

*Note* P-values of F-Test < 0.001 for both unadjusted and adjusted model. ^a^ Usability scores [23] range from 1 to 5; higher score = better usability. ^b^ UJACAS scores [10] range from 0 to 272, higher score = more active aging

In the unadjusted multivariable analysis with functional limitations, MOH was positively associated (*p* < 0.001) with active aging and a had positive moderating influence on the relationship between functional limitations and active aging (*p* = 0.045), indicating that attributing more meaning to the home, attenuated the negative influence of functional limitations on active aging. After adjusting for gender and education, the positive moderating influence of MOH on active aging remained (*p* = 0.039); education confounded the moderating influence, but gender did not. See Table 4 for details.

**Table 4:** Moderating influence of meaning of home ^a^ on the relationship between functional limitations and active aging ^b^, analysed using linear regression models, *N* = 820

Moderating influence of Meaning of home (MOH), dependent variable active aging			
Unadjusted model	Regression coefficient (β)	95% CI	*P*-value
No. of functional limitations	-3.7	-4.6; -2.8	< 0.001
MOH	1.7	1.1; 2.3	< 0.001
Interaction: MOH*No. of functional limitations	0.3	0.0; 0.5	0.045
R^2^ = 0.19			
Adjusted model			
No. of functional limitations	-3.3	-4.2; -2.4	< 0.001
MOH	1.7	1.1; 2.3	< 0.001
Interaction: MOH*No. of functional limitations	0.3	0.0; 0.5	0.039
Gender (Ref: Woman)	-2.9	-6.1; 0.2	0.065
Education (Ref: University 3 years or more)			
Education Elementary school	-15.6	-21.8; -9.3	< 0.001
Education 2 years upper secondary school	-6.4	-12.2; -0.7	0.029
Education 3 or 4 years upper secondary school	-4.1	-8.8; 0.5	0.079
Education University less than 3 years	-2.1	-6.2; 2.0	0.314
R^2^ = 0.22			

*Note P*-values of F-Test < 0.001 for both unadjusted and adjusted model. ^a^ MOH scores [15] range from 1 to 10, higher score = more meaning. ^c^ UJACAS scores [10] range from 0 to 272, higher score = more active aging

**Fig. 1:**
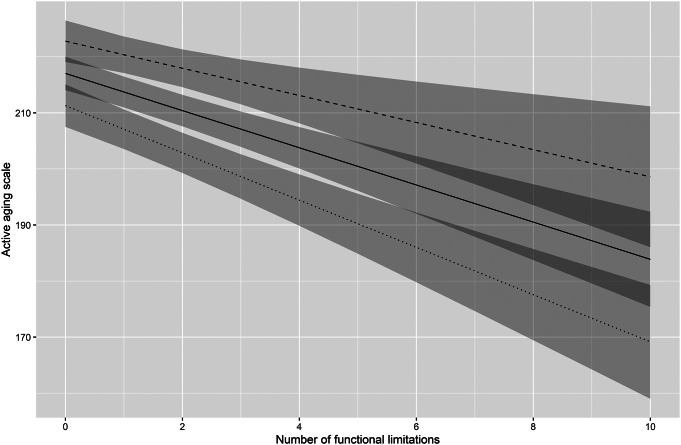
Illustration of the adjusted model analysis of a moderating influence of MOH on the relationship between functional limitations and active aging. *Note* The moderation influence was statistically significant (p=0.039). The solid line represents MOH mean, the dashed line MOH mean plus one standard deviation (SD), and the dotted line mean minus one SD. The light grey shaded areas visualize the 95% confidence intervals (CI) for the regression lines, with the overlaps of CI shaded dark grey

The moderating influence of MOH in the adjusted models on the relationship between functional limitations and active aging is illustrated in Fig. 1. For each functional limitation, those with high MOH had two points less decrease in active aging score compared to those with low MOH.

## Discussion

Starting out from the expected finding that active aging is inversely related to functional limitations, the main and novel contribution of this study is on the role of two different aspects of perceived housing on that relationship. While usability in the home does not moderate the relationship between active aging and functional limitations, MOH does. Thus, this study represents pioneering research addressing active aging empirically in the context of housing, with the potential to inform housing and health policies incorporating strategies and behaviors in daily life as suggested by the World Health Organization [34]. In addition, the study delivers knowledge about active aging among adults aged 55 + and considering relocation, showing that the self-rated level of active aging is high, particularly regarding self-assessed ability.

Reflecting on the main finding that MOH attenuated the negative relationship between functional limitations and active aging, we found that people with higher levels of MOH are able to maintain a higher level of active aging even with more functional limitations. Importantly, for people with lower MOH, active aging declined more rapidly with a higher number of functional limitations (Fig. 1). Knowing that very old adults who have stronger emotional ties to their home are more independent in daily activities [35], it is reasonable that MOH moderates the relationship we focused on. Reflecting on findings from studies involving younger older adults showing that behavioral aspects of MOH are related to more autonomy [20] and that the home becomes more important after retirement [36], in the present study, more perceived opportunity for activity could stem from the fact that people can and want to do more activities at home. As perceptions of MOH seem to change over time [26], it would be interesting to study how such changes moderate the relationship between functional limitations and active aging as people grow older. If reactive or proactive changes to the housing situation after retirement, for example, relocation to other forms of housing, would influence people’s perceptions of MOH, active aging might be affected positively or negatively. Because active aging as operationalized by the multi-dimensional UJACAS does incorporate psychological aspects, it could well be that having a home with more meaning gives a stronger incentive to be active despite declining functional capacity. As this was not explicitly teased out in our analyses using the total UJACAS score, further studies are warranted to shed light on such relationships. In addition, MOH is a psychologically complex concept, which might not even be possible to explain in the light of active aging. Summing up this reasoning, there is empirical support for considering the role of MOH further when studying active aging in the context of housing. Such knowledge is of theoretical as well as practical interest, not the least when it comes to housing policies and proactive housing counselling targeting younger older adults as they approach the phase of life when residential reasoning [37] and life transitions related to aging [29] emerge. The longitudinal design of Prospective RELOC-AGE is promising for forthcoming studies to follow the influence of MOH on the relationship between functional capacity and active aging over time.

Noteworthy, considering the interaction with MOH each functional limitation was associated with on average a decrease of the active aging score by three points, but the slope was markedly less steep among those with higher MOH (Fig. 1). To take a specific example, having two functional limitations, such as the fairly common reduced balance and stamina, compared to having none, high MOH lowers the associated decrease in the active aging score with four points. Reflecting upon the two strongest correlations between functional limitations and the active aging sub-scales found (data not shown in Results section: ability, *r*=-0.34, *p* < 0.001; opportunity, *r*=-0.29, *p* < 0.001), positive MOH may coincide with perceiving more opportunities for meaningful activities and the ability to do different activities.

While there are limited possibilities to relate to previous research, cautious comparisons can be made. Even if the UJACAS is not equivalent to an assessment of independence in activities of daily living (ADL; e.g., dressing, cooking, cleaning), but based on a specific definition of active aging [10] and including goals, ability and opportunity perspectives in addition to the traditional activity perspective, the scale nevertheless captures activity and participation. As the physical housing environment is not related to ADL independence among healthy individuals with few functional limitations but significantly among those with lower levels of functioning [17], it is reasonable to state that this facet of our current findings adds to the empirical support for the docility hypothesis [24]. According to this well-known hypothesis, the influence of the physical home environment (in our study operationalized as self-rated usability) on activity becomes more pronounced for people with compromised functioning. Considering that the mean age of our study sample was as low as 69.7 years with the vast majority in good health and with a very low prevalence of functional limitations, this finding is not surprising. Moreover, it should be noted that the usability questions address individual perceptions of the integration of person-environment fit and activity performance in the respondent’s current housing environment [25], but as this variable was only weakly related to the number of functional limitations (data not shown under Results: *r*= -0.16; *p* = 0.001) in our sample, it was not multicollinearity that led to no moderation. As functional capacity is integrated in the definition of usability [25], there could presumably not only be a direct effect from functional limitations on active aging (which includes a mix of in-home and out-of-home activities), but there could plausibly be an indirect effect through usability. Thus, usability might mediate rather than moderate the relationship between functional limitations and active aging. Because the distribution of functional limitations in our sample does not allow for sub-group analyses, we were not able to test such assumptions further at this stage. As this study is the first from Prospective RELOC-AGE addressing these intriguing dynamics, forthcoming follow-up data collections with this sample have the potential to shed further light on the role of the physical housing environment on relationships between functional capacity and active aging.

Even if minor, the statistically significant confounding effects of gender and education deserve comment. As to the confounding effect of gender in the adjusted regression model including usability, the confounding effect is not surprising because the usability items are related to ADL [25], and such activities are known to be gendered [38]. The confounding effect of educational level in both the adjusted models, indicating that education confounds the interactions of usability and MOH with functional limitations is intriguing. A plausible explanation based on practical experiences from data collection is that the MOH questionnaire can be challenging for respondents to grasp and comprehend because of its somewhat abstract and theoretically oriented content, which maybe could be harder for respondents with lower education to relate to. However, considering the very high educational level of our sample this is sheer speculation and warrants attention in future studies.

### Strengths and limitations

Overall, it might be seen as a limitation that we focused solely on the home environment, and not on neighborhood as an integral part of the concept of housing. Given the exploratory nature of the study and the complexity of the concepts included, this was a deliberate choice at this stage. Prospective RELOC-AGE includes a multitude of variables [31], including numerous neighborhood aspects that will be used in forthcoming studies from the larger project.

It should be kept in mind that Prospective RELOC-AGE has a multi-level mixed-methods design based on a recruitment strategy striving for an information-rich sample in the sense that participants were actively considering changing their housing situation [31]. While this is an asset given the overall objective of the project, it is a limitation because the sample is not representative of the general population aged 55 years or older. Rather, the sample was skewed towards healthy and higher educated people. The longitudinal design allows us to follow the participants as they age, which eventually will reduce the skewness in terms of health. The high proportion of higher educated people is a fact, and thus the findings must be interpreted with that in mind. Moreover, because the cross-sectional setting is a limitation when it comes to regression modelling involving interaction effects, the findings reported are at best indicative but valuable for future research on dynamics involving housing, health and active aging.

The descriptive results regarding active aging are well in line with previous studies using the UJACAS in population samples [10, 39]. However, it should be kept in mind that as is often the case, previous studies that displayed similar levels of active aging were based on samples different from the current study sample. In addition, given the limited body of existing research using UJACAS, there are yet no validated cut-off values for high versus low active aging.

Because two of the core instruments (i.e., MOH and UJACAS) were administered as part of the additional telephone interview to monitor reliability and validity, we had to use only a sub-sample (*N* = 820) of the Prospective RELOC-AGE baseline sample (*N* = 1,964) for the current study. However, as the sub-sample did not differ from the larger sample except from being slightly higher educated and including more women, this likely did not have any influence on the results. In fact, the use of telephone interviews was a strength as the participants had the opportunity to ask if they found some questions difficult to understand and get clarifying instructions as needed.

Reflecting upon the fact that the data collection was implemented during the COVID-19 pandemic, it should be kept in mind that it could have had an influence on the UJACAS data [40]. That is, the data collected were likely valid for the time of collection, but with a period effect that must be taken into consideration in future longitudinal analyses. Speculating further, because the home involuntary became the main arena for activities, the pandemic situation possibly affected MOH [41] and usability ratings as well. Forthcoming studies based on Prospective RELOC-AGE follow-up data will shed further light on such phenomena.

## Conclusion

This study on active aging in the context of housing shows that the expected inverse relationship between active aging and functional limitations is moderated by perceptions of MOH among healthy and highly educated older adults aged 55 + considering relocation, while perceived usability in the home does not moderate the relationship. Having a home with more personal meaning attached to it seems to provide more ability and opportunity for meaningful activities, thus supporting active aging despite functional limitations. This sheds new light on the previously known association between MOH and different aspects of wellbeing in old age. Using several complex concepts and scales in a cross-sectional study, the findings are of an exploratory nature and point to the necessity of further studies to tease out whether having a home with more meaning attached gives a stronger incentive to be active despite functional limitations. Results on perceived housing and active aging are of theoretical as well as practical interest and have the potential to nurture future research as well as housing policies and the development of housing counselling targeting younger older adults.

## Data Availability

The data used in this study contains sensitive information about the study participants and they did not provide consent for public data sharing, and the formal ethical approval does not include data sharing. A minimal data set could be shared by request from a qualified academic investigator for the sole purpose of replicating the present study, provided the data transfer is in agreement with EU legislation on the general data protection regulation and approval by the Swedish Ethical Review Authority.
